# Evaluation of BioFoam for Anastomotic Bleeding in Cardiovascular Surgery

**DOI:** 10.1055/s-0039-1678549

**Published:** 2019-02-15

**Authors:** Ottavio Alfieri

**Affiliations:** 1 Department of Cardiac Surgery, Ospedale San Raffaele, Milan, Italy

**Keywords:** BioFoam, hemostatic agent, surgical sealant, cardiovascular surgery

## Abstract

**Background** Hemostatic agents are increasingly used as an adjunct to standard methods of controlling anastomotic bleeding in surgical procedures. The purpose of this study was to investigate the safety and effectiveness of BioFoam Surgical Matrix used as an adjunct for anastomotic hemostasis following cardiovascular surgery.

**Methods** A prospective, multicenter, single arm study was conducted with 75 subjects treated with BioFoam following a total of 105 elective cardiovascular surgical procedures. Time to hemostasis was recorded following a single application of BioFoam in 74 subjects. Safety evaluations included intraoperative administration of a blood product, requirement for alternative means to achieve hemostasis, and the incidence of reoperation for bleeding.

**Results** Hemostasis within 3 minutes was achieved in 62 (84%) of the 74 subjects and within 10 minutes in 69 (93%) of these subjects. BioFoam was well tolerated. Twelve (16%) of the 75 enrolled subjects each experienced one adverse event, and 13 serious adverse events were reported in 10 (13.3%) of the subjects. None of the adverse events was considered by the Investigators to be related to BioFoam. Blood products were administered to 14 (18.6%) of the 75 subjects, banked autologous blood was given to 5 (6.6%) subjects, and 57 (75.7%) subjects required only a cell saver. Four (5.3%) of the 75 subjects required reoperation for bleeding within 24 hours of surgery. There were no observations of bleeding in any subject at discharge and no reoperation for bleeding following discharge. The mean operation time was 218.2 (±72.2) minutes.

**Conclusions** This study demonstrates the effectiveness of BioFoam Surgical Matrix when used as an adjunct for anastomotic hemostasis following a broad range of cardiovascular surgical procedures. The safety outcomes were within the normal limits for the types of procedures performed.

## Introduction

Cardiac and cardiovascular surgical procedures typically involve closure of the repaired or grafted tissues with sutures or staples which, despite careful application, can often leave small gaps through which blood may ooze slowly or profusely. Failure to rapidly and effectively control anastomotic bleeding can result in deleterious clinical sequelae that may include anemia, hemodynamic instability, hypothermia, hypovolemia, reduced oxygen delivery to tissues, impaired visualization of the surgical field, and an increase in the duration of surgery. 1 Moreover, uncontrolled bleeding can result in the need for a blood transfusion or reoperation and has been shown to be associated with an increase in the risk of mortality and a higher cost of treatment. 1

The common occurrence of anastomotic bleeding following cardiovascular surgery and the potentially grave consequences for the patient have driven the development and availability of an array of hemostatic methods. The methods range from simple application of manual pressure at the anastomotic site to the topical use of mechanical sealants and pharmacological agents. In addition to the application of pressure with a finger, sponge, clips, or sutures, other mechanical approaches include electrosurgery, laser, radio frequency energy, argon beam coagulation, ultrasonic scalpel, and ultrasonic surgical aspirator. 1 Sealants that create a physical barrier to staunch the flow of blood from the anastomotic site include gelatins, collagens, oxidized celluloses, synthetic glues, and glutaraldehyde-based glues. Thrombin and fibrin are the main pharmacological agents applied topically to promote coagulation of blood at the anastomotic site. 1 The choice of the hemostatic approach varies greatly between institutions and is generally based on the specific nature of the anastomotic site, the extent and speed of blood loss from the site, and the surgeon's experience and knowledge of available hemostatic methods. 2

Hemostatic agents have become established as a valuable adjunct to standard methods of controlling anastomotic bleeding in liver and spleen surgical procedures and are now increasingly used in cardiac and cardiovascular surgical procedures. The effectiveness of hemostatic agents in cardiovascular surgery has been reported in several clinical studies. In a prospective, multicenter, randomized study with 333 subjects, a fibrin sealant was found to control bleeding within 5 minutes of application in 92.6% of cases compared with only 12.4% of cases with the conventional agents. 3 Waragai et al reported a study with 112 subjects in which microporous polysaccharide hemostatic (MPH) bandage was compared with standard manual compression. Significantly fewer subjects treated with the MPH bandage required compression for 15 or more minutes ( *p* = 0.006) and significantly more subjects treated with MPH had a shorter time to hemostasis compared with compression alone ( *p* = 0.048). 4 In a study with 20 subjects undergoing carotid endarterectomy, the use of Quixil surgical sealant was found to greatly reduce the time to hemostasis ( *p* < 0.001) and the amount of blood loss ( *p* < 0.001) when compared with the use of a standard topical hemostatic agent. 5 These published studies and others have established a precedent for the use of hemostatic agents following a range of cardiovascular surgical procedures.

BioFoam surgical matrix further referred to here as BioFoam comprises two solutions contained separately in a double-barreled syringe: one barrel contained the protein bovine serum albumin (BSA) and sodium bicarbonate dissolved in water; the second barrel contained the cross-linking agent glutaraldehyde and acetic acid dissolved in water. BioFoam is generated when the plunger is pushed forcing the two solutions into a double-helix design tip which ensures their thorough mixing. The rapid reaction of sodium bicarbonate and acetic acid generates carbon dioxide which immediately expands the cross-linked BSA–glutaraldehyde complex into a flexible, mixed cell foam approximately three times the volume of the original solutions. Tests have shown that BioFoam has a burst strength of over 200 mm Hg which enables it to withstand postoperative hypertension. 6 The mixed-cell foam presents both a mechanical barrier to stop blood from oozing from the anastomotic site and a mixed-cell porous structure which allows entry of blood into the matrix where it aggregates. The presentation of BioFoam as preprepared solutions in a syringe that can be stored at room temperature enables its rapid deployment during surgery, while mixture of the solutions in the application tip ensures minimal wastage.

**Fig. 1:**
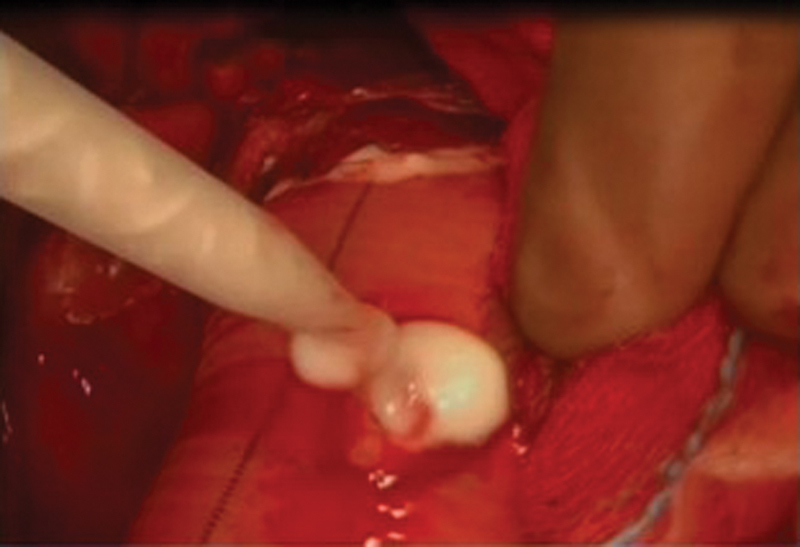
BioFoam on needle hole bleeding.

**Fig. 2:**
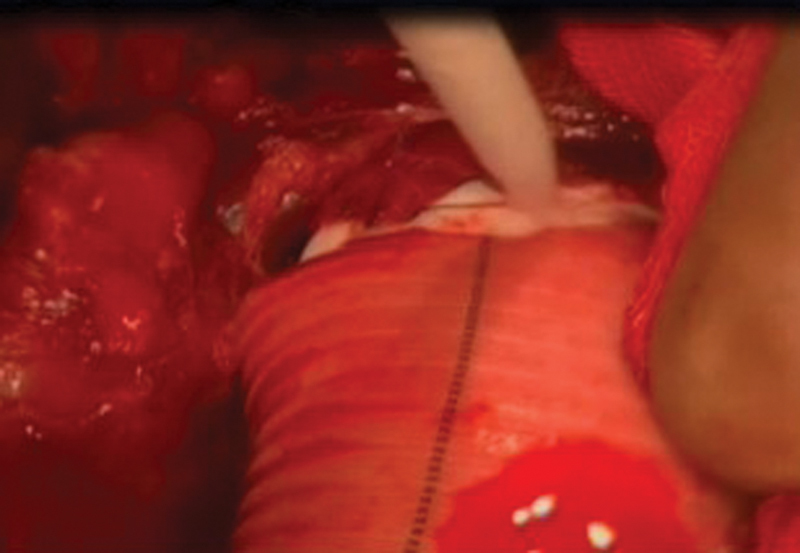
BioFoam on anastomotic suture line.

The present study was conducted to investigate the safety and effectiveness of BioFoam Surgical Matrix as a surgical adjunct to achieving anastomotic hemostasis following cardiovascular surgery ( Figs. 1 and 2 ).

## Materials and Methods

A prospective, multicenter, single-arm study was conducted to assess the safety and effectiveness of BioFoam used as a surgical adjunct to achieve anastomotic hemostatis following cardiovascular surgery. A total of 75 subjects were treated with BioFoam at two centers: 15 at the German Heart Centre, Munich, Germany; and 58 at the Ospedale San Raffaele, Milan, Italy.

The study was conducted in compliance with the protocol, the International Conference on Harmonization—Good Clinical Practice , and all applicable regulatory requirements. Participating investigating sites were responsible for complying with applicable regional or national regulations governing the conduct of postmarketing surveillance studies. The study was conducted in accordance with the World Medical Association Declaration of Helsinki adopted by the 18th WMA General Assembly in June 1964 and all amendments thereafter. The respective ethics committee of each clinical center approved the study protocol and all subjects provided their written informed consent before study enrolment. The study was listed on ClinicalTrials.gov (NCT02164201).

Patients included in the study were ≥ 18 years of age and scheduled to undergo an elective cardiovascular surgical procedure which could include but was not limited to thoracic aortic aneurysm, aortic valve replacement, and Type A aortic dissection (where the use of BioFoam was limited to the anastomotic site). Intraoperatively, the inclusion criterion was generalized oozing from the anastomotic repair site following use of standard repair procedures (such as sutures and staples) and for which the surgeon considered it necessary to use a hemostatic agent.

Exclusion criteria were: known hypersensitivity to albumin, bovine products, or glutaraldehyde; an active infection (either systemic or in the repair region); a pathology or underlying disease which made them unsuitable for clinical investigation as judged by the Investigator; a coagulation disorder; abnormal calcium metabolism (e.g., chronic renal failure or hyperparathyroidism); life expectancy less than that required for the follow-up duration; were pregnant, planning to become pregnant during the follow-up period, or actively breast feeding; or immunocompromised. No subjects were entered into the study on an “emergency use” basis. Subjects were also excluded if they experienced a major intraoperative bleeding incidence as defined by the American College of Surgeons Advanced Trauma Life Support Class II, III, or IV Hemorrhage.

The intent to treat population included all subjects enrolled and the per protocol cohort included subjects for whom there were no major protocol deviations including failure to meet any of the preoperative or intraoperative inclusion criteria, or any informed consent violation.

The hemostatic agent under investigation in this study was BioFoam Surgical Matrix, a combination of BSA and glutaraldehyde with foaming agents, as previously described. Investigators who were experienced and skilled in performing cardiovascular surgery were trained on the use of BioFoam per the Instructions for Use before participation in the study.

After completion of surgery, if a hemostatic agent was required to stop oozing from the anastomotic repair site, a temporary dry field was created with a swab or suction. BioFoam was then immediately applied to the anastomotic site and the region was closely observed at 1, 3, 5, 7, and 10 minutes. Time to hemostasis was recorded. All subjects were followed up for 30 days following surgery and assessed either during a patient visit to the hospital or by the surgeon directly speaking to the patient by phone.

The primary effectiveness end point was achievement of hemostasis (yes/no) of the repaired anastomotic site at 3 minutes after a single application of BioFoam. The secondary effectiveness end point was time to hemostasis assessed at 1, 5, 7, or 10 minutes (yes/no) after a single application of BioFoam ( Table 1 ). When hemostasis was achieved, observation was continued for a further 1 minute to confirm cessation of anastomotic bleeding. Immediate hemostasis was considered to have taken place when bleeding stopped within 1 minute after a single application of BioFoam and there was no oozing during a further 2 minutes of observation.

**Table 1:** Time to hemostasis

Time to hemostasis (min)	Number of subjects (%) *n* = 74 a
1	40 (54.1)
3	22 (29.7)
5	7 (9.4)
7	0 (0)
10	0 (0)
> 10	1 (1.4)
N/A b	4 (5.4)

a One subject was excluded as the time to hemostasis had not been recorded, although the achievement of hemostasis was reported for this patient.

b These subjects required reoperation for bleeding within 24 hours after the surgery. None of these events was considered by the Investigator to be associated with BioFoam.

The following assessments were made to evaluate the safety of BioFoam during this study: intraoperative blood product administration; use of alternative means to achieve hemostasis after application of BioFoam; the incidence of reoperation for bleeding; the total time of the operative procedure; the total hospitalization time; any additional hospitalization or surgical procedure through to the final follow-up period; the incidence of procedure complications; or adverse events (AEs) through to the final follow-up period. All AEs and serious adverse events (SAEs) were recorded and causal relationship with BioFoam was judged by the Investigator ( Tables 2 and 3 ).

**Table 2:** Adverse events

Type of event	Number of subjects (%) *n* = 75
Atrial fibrillation	11 (14.6)
Fever a	1 (1.3)
Bradycardia	1 (1.3)

a Patient with fever also required reoperation for bleeding.

**Table 3:** Serious adverse events

Type of event	Number of subjects (%) *n* = 75
Air in chest drain	1(1.3)
Left ventricular apex fissure a	1 (1.3)
Left arm ischemia a	1(1.3)
Reoperation for bleeding b	4 (5.3)
Pseudoaneurysm a	1(1.3)
Pericardial Effusion	1(1.3)
Wound Infection	1(1.3)
Asthenia and palpitations	1(1.3)
Sepsis	1(1.3)

a These events all occurred in the same patient.

b One patient returned to theater for bleeding on two occasions.

In addition to the standard measures of intraoperative status and the above-stated measures, the following measures specific to the use of BioFoam were recorded: estimated total amount of BioFoam applied; confirmation of proper coverage of the resected area with BioFoam; central venous pressure at the time of BioFoam application; and intraoperative blood loss.

## Results

A total of 95 subjects were screened and consented to participate in this prospective, multicenter, single-arm study. Of these 95 subjects, 20 (21%) did not meet intraoperative inclusion criteria. The remaining 75 subjects were treated with BioFoam. Two of the subjects at the German Heart Centre were lost to follow-up. As such, 73 subjects completed the study per protocol.

The baseline characteristics and medical history of the 75 subjects enrolled in the study are presented in Table 4 . All subjects were Caucasian.

**Table 4:** Subject demographics and medical history

Characteristics		Value (%)
Age		
Mean ± SD		67.91 ± 9.2
Range (min, max)		35–83
Gender		
Female		50 (66.6)
Male		25 (33.4)
Hypertension		53 (70.6)
Aortic aneurysm		20 (26.6)
Coronary artery disease		19 (25.3) 11 (14.7) Unknown
Smoking		41 (54.6)
Diabetes mellitus		10 (13.3)
Diabetes type		
Insulin dependent		4/10 (40.0)
Noninsulin dependent		6/10 (60.0)
Chronic obstructive pulmonary disease		12 (16) 1 (1.3) Unknown
Visceral occlusive disease		2 (2.7) 1 (1.3) Unknown
Chronic renal failure		3 (4)
Congestive heart failure		6 (8)
Cerebrovascular disease		3 (4)
Peripheral vascular disease		12 (16) 1 (1.4) Unknown
History of stroke		1 (1.4) 2 (2.6) Unknown
Malperfusion syndrome (thoracoabdominal)		3 (4)
NYHA functional class	1	29(38.6)
	2	25 (33.4)
	3	13 (17.3)
	4	6 (8)
	Unknown	2 (2.7)
Aortic insufficiency	None	25 (33.3)
	Mild	15 (20)
	Moderate	24 (32)
	Severe	9 (12)
	Unknown	2 (2.7)
Other relevant cardiac or vessel diseases		48 (64)

Abbreviations: NYHA, New York Heart Association; SD, standard deviation.

In total, 105 procedures were performed in the 75 patients. The types of surgery performed are shown in Table 5 . All subjects required the use of an adjunct hemostat to control generalized anastomotic oozing following standard repair procedures (such as sutures and staples).

**Table 5:** Type of surgery performed

Type of procedure	Number of subjects (%) *n* = 75
Mitral valve replacement	3 (4.0)
Redo mitral valve replacement	1 (1.3)
Redo aortic valve replacement	1(1.3)
Aortic valve replacement	27 (36)
Aortic valve replacement with CABG	7 (9.4)
Aortic valve replacement with aneurysm repair	10 (13.5)
Aortic valve replacement with mitral valve replacement	5 (6.8)
Aneurysm repair	10 (13.4)
Aortic stenosis	1 (1.3)
Aortic valve replacement + aneurysm repair + CABG	1 (1.3)
Aortic valve replacement + atrial fibrillation ablation	1 (1.3)
Mitral valve replacement + tricuspid valve	1 (1.3)
Mitral valve replacement + tricuspid valve replacement + aortic repair	1 (1.3)
Mitral valve replacement + tricuspid valve replacement + ACVB	1 (1.3)
ACVB + left ventricular aneurysm	1 (1.3)
ACVB	1 (1.3)
Mitral valve replacement + ACVB + atrial septal aneurysm	1 (1.3)
Mitral valve annuloplasty + LAA occlusion	1 (1.3)
Aortic valve replacement + marrows myomectomy	1 (1.3)

Abbreviations: ACVB, aortocoronary venus bypass; CABG, coronary artery bypass graft; LAA, left atrial appendage.

The time to hemostasis was measured in 74 of the 75 subjects treated with BioFoam. Of these 74 subjects, 62 (84%) met the primary effectiveness end point of hemostasis achievement within 3 minutes. The secondary effectiveness end point of hemostasis within 10 minutes was achieved in 69 (93%) of the 74 subjects. The results for primary and secondary effectiveness end points are shown in Table 1 .

Hemostasis was achieved after 10 minutes in one of the five subjects who did not achieve the secondary effectiveness end point. Of the remaining four subjects, one required the use of additional sutures; two required the use of alternative adhesives—Tachosil was applied in one case and BioGlue in the other; and no additional procedure or reintervention was recorded for the fourth subject.

Less than 5 mL of BioFoam was used in 72 of the 75 subjects (96%) treated with BioFoam. In 74 (98.6%) of the 75 subjects, the volume of BioFoam applied was considered by the Investigator to have adequately covered the oozing area. BioFoam did not adequately cover the surgical site in one (1.3%) of the 75 subjects. In this subject, although 10 mL of BioFoam was applied to the left atrial appendage during mitral valve repair over a tissue to tissue anastomosis, the surgeon noted that the BioFoam did not adhere to the tissue. Tachosil was applied to the tissue but hemostasis could not be adequately maintained and the subject underwent reoperation for bleeding.

Overall 12 (16%) of the 75 subjects experienced one AE ( Table 2 ) and 13 SAEs occurred in 10 (13.3%) of 75 subjects ( Table 3 ). The most common AE reported was atrial fibrillation which occurred in 11 (14.6%) of the 75 subjects. None of the AEs or SAEs reported in this study were considered by the Investigators to be related to the use of BioFoam.

Four (5.3%) of the 75 subjects required reoperation for bleeding within 24 hours of surgery. None of these events was considered by the Investigator to be associated with an area of BioFoam application. There were no observations of bleeding in any subject at discharge and no reoperation for bleeding following discharge.

Blood products (red blood cells, platelets, and fresh frozen plasma) were administered to 14 (18.6%) of the 75 subjects. A further 5 (6.6%) subjects were given banked autologous blood and the majority, 57 (75.7%) of the 75 subjects, required only a cell saver. A mean operation time of 218.2 (±72.2) minutes was observed in this study.

## Discussion

Patients who undergo cardiovascular surgical procedures which have been repaired with sutures or staples often have anastomotic bleeding that must be rapidly and effectively controlled by the surgeon to avoid deleterious clinical consequences. The clinical sequelae may include anemia, hemodynamic instability, hypothermia, hypovolemia, reduced oxygen delivery to tissues, impaired visualization of the surgical field, an increase in the duration of surgery, blood transfusion, and reoperation. 1

BioFoam has been developed to offer surgeons a new option for achieving rapid and effective anastomotic hemostasis—with a convenient prefilled syringe that can be stored at room temperature. Initially registered in 2009 for use in liver and spleen surgery, BioFoam received approval in 2012 for an extended indication in cardiovascular surgery. The convenient packaging that obviates the need for preparation prior to use and the stability of BioFoam at room temperature are intended to enable rapid deployment. 7 Speed of application of the hemostat can be an important consideration when bleeding is profuse and the patient's prognosis is under threat.

This prospective, multicenter, single arm study demonstrated the safety and effectiveness of BioFoam as a surgical adjunct to achieve anastomotic hemostatis following cardiovascular surgery in 75 subjects at two centers (15 subjects at the German Heart Centre, Munich, Germany, and 58 subjects at the Ospedale San Raffaele, Milan, Italy). The primary effectiveness end point of hemostasis achieved within 3 minutes was observed in 62 (84%) of the 75 subjects ( Table 1 ). Hemostasis was achieved in a further five subjects within 10 minutes after application of BioFoam. The effectiveness of BioFoam in a broad range of cardiovascular surgical settings in this study supports its general use for addressing anastomotic bleeding following cardiovascular surgery.

BioFoam was well tolerated in this study. None of the AEs in 12 (16%) subjects or the 13 SAEs in 10 (13.3%) of the 75 subjects were considered by the Investigator to be related to the use of BioFoam. Four (5.3%) of the seventy-five subjects required reoperation for bleeding within 24 hours of surgery. The safety outcomes for this study were comparable to those observed in studies with established surgical sealants used in cardiovascular surgery. 8

Blood products (red blood cells, platelets, and fresh frozen plasma) were given to 14 (18.6%) of the 75 subjects and a further 5 (6.6%) required banked autologous blood. The majority of subjects—57 (75.7%) of the 75 subjects—required only a cell saver. A low requirement for blood products, such as that observed in this study, has been shown to be associated with better outcomes for patients. 9

The mean operation time of 218.2 (±72.2) minutes observed in this study is similar to that observed with BioGlue, a nonfoaming formulation sealant produced by CryoLife. 10

It is acknowledged that this study does have limitations. As this was not a randomized study, there is no comparison to any “other agent” or a comparison to “no agent,” so although the results are promising, there is no direct proof of superiority versus other methods of controlling bleeding. While protamine was administered to most of the patients during surgery, use of protamine before or after BioFoam application was not recorded. As such, it is not possible to ascertain the possible contribution of protamine to cessation of bleeding in this study.

Overall, the results of this study support the safety and effectiveness of BioFoam Surgical Matrix when used as an adjunct for anastomotic hemostasis following cardiovascular surgery.
